# A Qualitative Study on Sexuality and Sexual Experiences in Community Forensic Mental Health Patients in Queensland, Australia

**DOI:** 10.3389/fpsyt.2022.832139

**Published:** 2022-03-31

**Authors:** Elnike Brand, Dinesh Nagaraj, Angela Ratsch, Edward Heffernan

**Affiliations:** ^1^Faculty of Medicine, The University of Queensland, Brisbane, QLD, Australia; ^2^Community Mental Health and Addiction Services, Waikato District Health Board, Hamilton, New Zealand; ^3^Wide Bay Hospital and Health Service, Research Services, Hervey Bay Hospital, Hervey Bay, QLD, Australia; ^4^Rural Clinical School, The University of Queensland, Brisbane, QLD, Australia

**Keywords:** forensic order, schizophrenia, sexual health, sexuality, major mental illness

## Abstract

This qualitative study reports on the sexuality and sexual experiences of community-based forensic mental health participants. The findings indicate that these participants feel the need for intimacy and want to engage in sexual activity more often than their neurotypical Australian peers. Participants identified their mental health and the side effects of compliance with prescribed psychotropic medications were barriers to achieving their desired level of sexual activity. Participants supported the notion that mental health teams were positioned to assist patients navigate the psychological, cultural, education and physical barriers to achieving sexual health and wellbeing. We propose several interventions to support these participants and other community forensic mental health patients in attaining healthy relationships, understanding their sexual health, and gaining more fulfilling sexual experiences. These interventions, which include sex education, upskilling in socialization and communication, and regular medication reviews, could be delivered as part of the holistic care provided by mental health teams. Mental health clinicians should be offered appropriate training to assess patients and have discussions related to sexuality, sexual experiences and sexual health needs.

## Introduction

“*Those of us who have been diagnosed with major mental illness do not cease to be human beings by virtue of that diagnosis. Like all people, we experience the need for love, companionship, solitude, and intimacy. Like all people, we want to feel loved, valued, and desired by others.”* (1)

“Sexuality” can be understood as a central aspect of being human. Throughout life, it encompasses sex, gender identities and roles, sexual orientation, eroticism, pleasure, intimacy and reproduction (2). Sexuality is experienced and expressed in a multitude of ways, such as in thoughts, fantasies, desires, beliefs, attitudes, values, behaviors, practices, roles and relationships (2). The expression of sexuality can be impacted by various considerations, including psychological, social, cultural, legal and religious influences (3). Sexuality and sexual functioning are aspects of an individual's sexual health and are essential components of the individual's holistic health (4), with most people feeling the desire to have an intimate relationship and to engage in sexual activity, including those with mental illness (5). The World Health Organization (2) defines sexual health as:

a state of physical, emotional, mental and social wellbeing in relation to sexuality; it is not merely the absence of disease, dysfunction, or infirmity. Sexual health requires a positive and respectful approach to sexuality and sexual relationships and the possibility of having pleasurable and safe sexual experiences, free of coercion, discrimination, and violence.

### The Intersection of Sexology and Mental Health

Most major mental illnesses are chronic and have significant impacts on all aspects of life, including sexual health, sexuality and sexual functioning (6). Studies (7, 8) show that once an individual has been diagnosed with a major mental illness, their mental healthcare becomes the overwhelming focus of their clinical care, circumventing their sexual healthcare needs. This healthcare deficit becomes exaggerated for people diagnosed with a forensic mental health disorder, extending to an absence of services (9). This gap in sexual healthcare is surprising given that there is a concerted focus on health generally in mental health recovery and rehabilitation (10, 11).

In the practice of mental health, there appears to be a clinical knowledge, assessment and treatment gap firstly around sexology, and then at the intersection of sexology and mental health (12). Healthcare workers often lack awareness and understanding of their patients' sexual health, sexual function, sexual knowledge, and sexual practices (13). In addition, as Dyer and das Nair (14) have found, clinicians are generally unaware of the depth of impact on sexual function that many mental health conditions and treatments can potentially have, including current and future relationships and commitment to treatment (15).

Psychotropic medications are directly associated with sexual dysfunction, decreased libido, erectile dysfunction, anorgasmia, and decreased ejaculatory volume (16). These adverse effects have a considerable impact on quality of life and contribute to patients' decisions to adhere to psychotropic medications (17). Adherence to psychotropic medications has downstream consequences, perpetuating the problem of sexual dysfunction such that it manifests as a bi-directional sexual health-mental health connection (17–19).

### The Challenge

The first Australian large-scale national data study on the sexual health of Australians [*Australian Study of Health and Relationships 2* (20)] provided robust information around sexual health, experiences, knowledge and education in the general Australian population. However, sexuality is unique in the created tension—it is both mundane and exciting (21). On the one hand, it is a part of everday normal life experiences. On the other, it is regarded as a sensitive topic and certainly a clinical assessment criterion that can create discomfort for clinicians (14) and patients (22). This is possibly why very few studies have evaluated sexuality and sexual functioning in people with mental illness, even though the evidence shows that mental illness, social impairments, and institutionalization all affect an individual's sexuality and sexual functioning (23). The first aligned Australian study on the subject was conducted with prisoners from New South Wales and Queensland (*n* = 2,351) and examined their sexual health and behavior (24). That study was followed by McMillan et al. (7) who examined some aspects of sexual functioning and experiences in young people affected by mental health disorders (*n* = 103). Both studies provide useful information in their discreet field, however there is an absence of data on the sexual health in people experiencing a major mental illnesses and how that diagnosis impacts on their healthcare including their sexual healthcare requirements.

### Aims

The aim was to qualitatively examine the sexuality and sexual experiences of community forensic mental health participants treated under the requirements of a forensic order.

## Methods

### Setting

The study was conducted from January 2020 over 3 months across several community mental health centers in Queensland, Australia, including rural, regional and metropolitan areas.

### Design

This is the third phase of a larger research program that investigated forensic mental health participants' sexuality and sexual health needs, the background and protocol of which is published elsewhere (21, 25). This qualitative phase was focused on providing participants an opportunity to talk openingly with researchers whom they were now familiar with, and for the researchers to better understand the participants current sexual experiences, future expectations in terms of sexuality, their barriers to achieving their ideal sex life, and the perceived need for involvement by their mental health team.

### Sample

The population included people with a major mental illness who were mentally stable, community-based, and treated under the requirements of a Forensic Order (Queensland) (26). [A Forensic Order is made by the Mental Health Court for persons charged with a serious offense who are found to be of unsound mind at the time of an alleged crime or unfit for trial. Persons on a Forensic Order have specific legal requirements to attend mental health care and are under the close care of an authorized doctor and mental health team. The team provide a very structured and supportive clinical network to ensure and enable the patient to engage in recovery and rehabilitation. This team, which includes a Forensic Liaison Officer (FLO), is responsible for providing appropriate care and managing treatment.] The sample consisted of 14 purposely enrolled information-rich participants: 11 males and 3 females from a sample of 50 potential participants. This sample was identified as information-rich from Phase 1 and Phase 2 [*n* = 235 potential participants (25)]. Patton (27) explains that the logic and power of purposeful sampling is in identifying and selecting individuals who are particularly knowledgeable about or experienced with the phenomenon of interest, thus the terms purposeful sampling and information-rich participants. In addition to knowledge and experience, purposely enrolled participants need to be available, be willing to participate, and have the ability to communicate experiences and opinions in an effective manner (28).

Potential participants in this phase that were deemed by the FLO as being vulnerable to distress, such as participants with recent exposure or disclosure of sexual abuse or assault, had been excluded in Phase 1 and 2 (Table 1), as had potential participants with significant cognitive impairment, including intellectual disability. All participants completed an informed consent form provided by their FLO. The research team assessed the participants' capacity to consent and their mental state before the interviews, during, and after the interviews.

**Table 1:** Inclusion and exclusion criteria.

**Inclusion criteria**	**Exclusion criteria**
≥18 years Diagnosed with a major mental illness Receives treatment under the requirements of a Forensic Order Capacity to consent and participate Community based	IQ < 70 Vulnerable people—recent exposure or disclosure of sexual abuse or assault Currently hospitalized for mental illness

### Data Collection

The current study was the third phase of a larger research project and was conducted over a 3-month period from January 2020. The 14 participants' demographic details were collected from their pre-assessment using a standardized form. The participants involved in the study were interviewed by the same forensic psychiatrist who conducted Phase 1 and 2 data collection interviews. The intent was to provide a familiar researcher in an environment familiar to the participant and conduct the interviews in a familiar format in order to foster open dialogue. The interviews took place in the same community forensic mental health clinics that participants typically received their clinical care from the community forensic team. Participants were given the option to have a support person with them during the interview process.

Interviews were face-to-face and informal, and were conducted using a semi-structured, open-ended questionnaire (Appendix 1) to allow for flexibility with varied depth. This approach allowed participants to share their experiences and understanding using their own words. The interview covered questions regarding their current sex life, level of satisfaction, their ideal sex lives and barriers that have prevented them from achieving this, and the potential role of their mental health team. Each question had further prompts to encourage participants to elucidate and uncover a deeper meaning of sexuality as expressed by each participant in their thoughts, fantasies, desires, beliefs, attitudes, values, behaviors, practices, roles and relationships. The interviewer encouraged participants to discuss any concerns, distress, or discomfort with the interviewer at any time.

Each interview lasted for about 30 min and was audio recorded. At the end of the interview, participants were given a feedback form to comment on their perceived embarrassment with the interview process and the level of transparency in their responses. Both these details were captured on ten-point Likert scales. Participants did not have the opportunity to review or edit their responses after completing the interview. Data collection ceased at 14 participants as saturation was reached.

### Data Analysis

The recorded interviews were transcribed verbatim by a medical transcriber. The interviews were analyzed using Braun and Clarke's (29) thematic analysis method with the researchers identifying the main themes and noting any similarities and differences between participants. Common concepts were extracted from the transcribed interviews. These concepts were coded and further classified into themes and subthemes by two independent researchers. After the initial themes were generated, they were reviewed again 1 week later and separately coded. These two sets were then compared against each other, further improving the final set of themes. The themes and subthemes were compared and condensed if found to be similar.

## Results

The participants ages ranged from 25 to 61 years old (mean = 45.14, standard deviation = 11.90). All 14 participants opted not to have a support person during the interview. Ten of the participants had a primary diagnosis of paranoid schizophrenia, one had schizoaffective disorder, one had undifferentiated schizophrenia, one had bipolar affective disorder and one had unspecified psychosis. Table 2 shows that none of the participants were in a long-term relationship; nine were never married, two were separated and three were divorced.

**Table 2:** Participant details.

**Variable/sex**		**Male** **(*n* = 11)**	**Female** **(*n* = 3)**	**Total** **(*n* = 14)**
Age (years)		Range = 25–61	Range = 37–46	Range = 25–61
		Mean = 45.72	Mean = 41.67	Mean = 45.14
Education	Junior education	1	0	1
	Junior secondary education	6	3	9
	Secondary education	2	0	2
	Unspecified	2	0	2
Marital status	Never married	7	2	9
	Separated	1	1	2
	Divorced	3	0	3
Primary diagnosis	Paranoid schizophrenia	7	3	10
	Undifferentiated schizophrenia	1	0	1
	Schizoaffective disorder	1	0	1
	Bipolar affective disorder	1	0	1
	Unspecified psychosis	1	0	1

The Australian educations system structures schooling from Prep to 12. Primary school consists of 6 years from Year 1 to 6; secondary school consists of 4 years from Year 7 to 10; senior secondary school consists of years of Year 11 and 12. One participant had not completed past Year 6, nine had not completed past Year 10, two had completed year 12, and two were not able to comment on their level of schooling.

On the Likert scales (on a scale on 1 to 10, where “0” indicated “not at all” and “10” indicated “a high level”) that measured the participants' level of embarrassment with the interview process, all 14 of the participants marked a “0”, indicating that they did not feel embarrassed. With regards to their level of transparency, all 14 participants had marked a “10” on the Likert scale, highlighting that they were open and transparent in their responses.

Three themes were identified in the analysis.

### Theme One: Participants Were Not Sexually Active, but Eager to Be in a Sexual Relationship

“*I don't have anyone, I am on my own … I do still like to get married one day”—Participant 1, Male, 58*

Of the 14 participants who were interviewed, only two of them were currently partnered and had a stable relationship. The other participants had been single for periods upwards of 6 months. Most participants had commented that they were happy and satisfied with their current situation of not being active sexually, but on further exploration, it did come to light that they missed the intimacy in a relationship.

“*I don't just want to go ahead and have sex for the sake of having sex, no, it's not my cup of tea … a good relationship is where you can help each other.”—Participant 4, Male, 61*

All the participants interviewed were not sexually active, all of them except two participants had indicated that they would prefer to have sexual relationships; these two participants were not interested in the sexual component of a relationship at all, citing that their need for companionship, stability, and marriage was more significant than their need for sexual activity. Subthemes identified included, the participants *were more seeking for emotional intimacy rather than sexual intimacy* and, participants harbored *a lack of motivation to look for or pursue a relationship*.

All participants ranked highly their need for a stable, romantic relationship that provided companionship and intimacy. These factors were perceived as more important than the relationship, including sexual activity. None of the participants reported wanting casual partners or casual sex. Some participants reported a desire to wait until marriage to begin sexual activity. Twelve of the fourteen participants reported a desire for sex with a regular partner. Their desired ideal of sexual intimacy ranged from having sex at least twice per week to having sex daily (an average of 3.71 times per week).

Four of the 12 single participants stated that they were not actively searching for a partner and lacked the motivation to do so, while others have had significant difficulty in their search.

“*I want to be in a stable relationship, but I am not really trying at the moment …”—Participant 11, Male, 33*

### Theme Two: Mental Health and Medications Were Barriers to Achieving Fulfilling Relationships and Sexual Experiences

“*I think one of my major problems is my mental health. I was diagnosed when I was 20 … the period in life when people meet each other and everything … I was living with my parents then.”—Participant 3, Male, age 33*

Twelve participants identified their mental illness and medications as barriers to achieving fulfilling relationships and sexual experiences. These participants have long-standing, major mental health issues, with most participants holding a diagnosis of paranoid schizophrenia. Many had been exposed to psychotropic medications and the well-known sexual side effects. While some participants recognized and acknowledged the positive effects medications have on their mental state, they also recognized this came with a detriment to their sexual function. One participant highlighted the fear of getting hurt again based on his experience of a relationship breakup. One participant could not identify potential barriers to achieving fulfilling sexual experiences. The subtheme of *difficulties in socializing and communication* was observed.

“*I just stay home most of the time; don't get out,… I think it's because of my illness.”—Participant 7, Male, 55*

For those who wanted to look for a regular partner, challenges in socializing and communication were reported as significant barriers. They attributed their mental health symptoms or past institutionalization to creating social tension. One participant indicated that, following years of institutionalization, he was not computer literate and therefore unable to use modern technology to connect with a partner.

### Theme Three: Participants Supported the Notion That Mental Health Teams Could Support Their Sexual Health and Wellbeing

“*I think the mental health team can improve my relationship but not sexual-wise, yeah. You know, more about communicating and all that probably.”—Participant 7, Male, 55*

There were varying degrees of consensus about the role of a mental health team in participants' sexual wellbeing. Five participants either reported that mental health teams had no role to play or were uncertain how their mental health teams could support them. Of those that said there was no role for mental health teams, three agreed that the teams would be helpful for other patients. The remaining nine participants had a wish for their mental health teams to play a role in supporting their sexual wellbeing. They indicated no issues with being asked questions regarding their sexual health and wellbeing during regular reviews. Some were able to identify the areas in which they would like to receive aid, such as knowledge about what “rights” they would have in a relationship, working on their communication skills, and regular medication reviews to maximize the treatment effect while minimizing side effects.

## Discussion

This qualitative study describes the sexuality and sexual experiences of 14 participants who were enrolled as patients in a community forensic mental health service. This study took place in the early months of 2020 and is a reflection of the participants' experiences prior to the impact of the COVID-19 pandemic. At the end of the interview, all 14 participants rated high on Likert scales pertaining to the transparency of their given responses. They rated low on the level of discomfort and embarrassment in engaging in the current qualitative study that explored sensitive topics. This provides an impression that the themes identified in the current study have a high degree of validity and transferability when it comes to generalizing these findings of sexuality and sexual experiences on other community forensic mental health patients treated under a forensic order. However, more studies need to be carried out in community forensic settings to confirm the presence of similar themes in this cohort of patients. Given the complexity and heterogeneity of forensic mental health patients, there is a significant difference from patients with severe mental illness without a forensic history, thus the results from this study cannot be applied to mental health patients without a forensic history. In the same line of argument, the qualitative findings illustrated in this study in Queensland, Australia, cannot be generalized across other countries because of differences in norms, expectations, values, attitudes, beliefs and behaviors regarding sexuality from societal, cultural and religious standpoints. Findings from future qualitative studies conducted in other countries will help identify anthropological differences across various ethnic groups, religions and cultures and support the health practitioner to be more culturally sensitive when evaluating patients within their own countries, as well as inform the care of migrant patients.

Most of the participants were single, and none were sexually active. Eleven of the fourteen participants reported that they were satisfied with the lack of a current sexual partner, while only three indicated their dissatisfaction. However, all participants voiced their hopes for a stable partner, and 12 wanted regular sex with that partner.

“Regular sex” for participants was defined as ranging from two times per week to every day, with an average of 3.71 times per week. This number is higher than the normative Australian population, where people with regular partners had sex at an average of 1.4 times per week (20). The increase in the need for sexual intimacy amongst participants might indicate that mental health patients have a higher sex drive than the average Australian, despite the fact that psychotropic medications can reduce libido (30). Another possible explanation is that this is a display of hoarding; similar to hoarding food during physical starvation, participants display an increased desire for sexual intimacy when unavailable (31).

A notable finding was that participants reported a high level of satisfaction with their current level of sexual activity despite the incongruence with their desired level of sexual activity. This discrepancy could be explained by the phenomenon of “learned helplessness,” a term coined by Seligman (32, 33) after his experiments in which he subjected canines to uncontrollable shocks. He concluded that with enough trials of subjecting organisms to noxious stimuli from which they cannot escape, eventually, the organism will stop trying to escape even if the opportunity presents itself. “Learned helplessness” could explain the participants' view on their inability to escape singledom. Negative experiences with relationships, and the pursuit of relationships, in the past, have caused an acceptance of their situation and learnt contentment.

Another possible explanation of this finding is that the participants are showing denial of the situation, a concept first described by Freud (34). This denial is described as a defense mechanism used by individuals to avoid the situation which causes them anxiety. By refusing to acknowledge a negative situation, the negative emotions associated with the situation can also be avoided. This explication is supported by some statements made by participants that they had not thought about sexual relationships for some time. This lack of thought, and acknowledgment of the current lack of sexual intimacy, could explain the perceived level of contentment.

Another notable finding was participants' lack of desire for casual sex. The desire for sexual activity was entirely overshadowed by a desire for the stability and intimacy that come with a serious romantic relationship. This finding could be explained using the modified version of Maslow's Hierarchy of Needs proposed by Kenrick et al. (35). Their model uses evolutionary theory to update the traditional hierarchy of needs. This model places the need for affiliation before the need for mate acquisition. This theory supports the participants' statements, as the need for social support and belonging will take priority over finding a sexual relationship, according to this model.

A different interpretation that can be posited from the participants' notions of being satisfied with their current state of being sexually inactive, despite the incongruence with their desired level of sexual activity, and the aspect of lacking the motivation to pursue a relationship can be explained as a phenomenon of these people to have lost a sense of hope on matters of relationships and intimacy. Most people with an enduring mental illness will feel disempowered after being subjected to years of treatment under a legal framework, involuntary confinement, and experiences of other treatment interventions that they might perceive as a paternalistic approach (36). In the process of their treatment trajectory, patients lose hope and conform to an image of worthlessness and incapacity, becoming socially withdrawn and adopting a disabled role (37). All this could lead to the abnegation of previously held ambitions and values, including those pertaining to sexuality and intimacy.

Unfortunately, forensic mental health patients face many barriers in searching for romantic and sexual partners. These patients often have long-standing major mental illnesses that usually begin to manifest in the teenage years. This period coincides with sexual development (38). Healthy sexuality is a critical developmental milestone (39, 40), that mostly happens in adolescence (41), a time when there is also an increase in mental health incidence (42). Sexual dysfunction caused by medications and its psychological impact on individuals at a young age has the ability to thereby affect their sexuality (7).

All participants in this study have had severe mental illnesses throughout their lives and have been exposed to a multitude of psychotropic medications, including antipsychotics, antidepressants, and mood stabilizers over prolonged periods which impacted their ability to initiate and sustain intimate relationships (43). The participants in the study did not report any difficulties in memory, attention or other cognitive deficits. However, from the interview process and judging the answers given, there is a possibility that there were underlying cognitive deficits. Many participants gave monosyllabic replies, had a disjointed thought process, and answered in a tangential manner. The cognitive deficits (44) and sexual side effects of these medications are well documented. These cognitive deficits, especially in the prefrontal lobe area, can impact insight and judgment. In the context of this study, this could affect the participants' abilities to formulate the steps needed to achieve their desired romantic and sexual goals.

The academic literature identifies the barriers clinicians face when engaging in discussions with their patients around sexuality and intimacy. This could be due to embarrassment, fear (14), or the belief that discussing sexual activity could damage the therapeutic relationship (45). This likely contributed to participants' uncertainty on how their mental health teams can support them in achieving fulfilling sexual experiences. Most participants were open to their mental health teams asking questions about their sexual experiences. Some participants were able to specify precisely the areas they would like to be addressed by the mental health team: mainly sex education, communication skills, and medication reviews. Assalian, Fraser (46) concur that sex education can enhance confidence and knowledge around partnership and healthy sexual relationships.

## Recommendations

A literature review had demonstrated significant gaps in the sexual health and wellbeing of people with severe mental illness. Compared to other health needs such as drug and alcohol addiction, domestic violence, and rehabilitation, there is a lack of support groups for those whose sexuality has been negatively affected by mental illness. There is also an absence of any evidence-based, individualized approaches which clinicians can use to help mental health patients achieve a healthy sexual life (47).

Forensic mental health patients would benefit from support to achieve better sexual experiences to improve their quality of life. To support the patients in this regard can be a service delivery role that mental health teams can offer through: sexual education programs (16, 23, 48–51), improvement of socialization and communication skills (8, 52–54) and address issues of internalization of stigma and self-esteem (40, 54–59), and medication reviews (47).

Sex education, incorporated as part of standard rehabilitation, might address inappropriate sexual behaviors while reducing social and sexual isolation (18, 23, 49, 53, 60–67). Social isolation is a known risk factor for relapse of schizophrenia, which has a negative effect on patients' abilities to integrate into their community once released from institutions (49). Deprivation and isolation from sexual activity and tactile affection correlate with several negative emotions, such as loneliness, depression, stress, and alexithymia (68).

Communication and social skills training will require input from allied health professionals (49, 51, 67, 69, 70). Mental health patients can be emotionally stunted and have underdeveloped social skills, leading to their inability to form and maintain relationships (46). Thus, early intervention, especially during early psychotic episodes, might prevent social isolation, improve quality of life and ensure more successful reintegration into the community (71). Integrating programs focusing on the development of intimacy and healthy sexual relationships can reduce social and sexual isolation (46).

Mental health clinicians have cited lack of training as their primary barrier in initiating discussion regarding sexuality (11, 14). Medical education often does not adequately prepare clinicians for in-depth discussions about their patients' sexual health (72–74). The inadequacy in training results in clinicians feeling uncomfortable broaching the topic and therefore avoiding doing so (14, 75). All clinicians are taught the basics of a successful clinical interaction: personalized information, active listening, checking comprehension, establishing a safe environment, assessing readiness for change and reassurance (76). Using these tools, healthcare workers should be able to start a conversation around patients' sexuality, sexual experiences, and sexual wellbeing. This approach will maximize the potential of a successful outcome of a personalized intervention strategy.

The sexual side effects of psychotropic medications, including antipsychotic medications, are well recognized (30) with typical antipsychotics more likely to cause extrapyramidal side effects and sexual dysfunction compared to atypical antipsychotics (17, 23). According to Assalian, Fraser (46), many psychiatrists attribute patients' reported sexual dysfunction to internal, unconscious conflict rather than medication side effects (77). Significant discordance is evident between patient reports and psychiatrist perception of sexual dysfunction, with psychiatrists underestimating sexual dysfunction (78, 79). Iatrogenic sexual dysfunction is mostly reversible with dose reduction or termination with substitute medication (80). Psychiatrists should consider the lowest effective dosing (16, 23, 81), monotherapy, prolactin-sparing antipsychotic medications in the management of psychotic illness with consideration to sexual side effects (17, 82, 83). Regular medication reviews can help maximize treatment effects while minimizing side effects for these patients. Appropriate choice of medications are crucial because iatrogenic sexual dysfunction can reduce medication compliance and compromise therapeutic relationships (46).

## Strengths and Weaknesses

This study investigated a significant gap in the literature and collected robust qualitative data from forensic mental health participants. The study was cross sectional and enrolled participants who had originated from all areas of Australia, and who were now residents in Queensland. Less women were enrolled in the study, such is the normal profile of forensic order patients. The findings are important to understand the sexuality and sexual experiences of this marginalized group and highlight significant barriers for participants to achieve their desired goals pertaining to their sexuality; this information will help mental health teams to formulate necessary interventions to support community forensic mental health patients on matters of their sexuality and sexual health. Additionally, this study shed light on participants' perception of mental health teams' role in supporting their overall sexual health. These findings will inform clinical practices pertaining to supporting sexuality and sexual health needs of community forensic mental health patients.

The study was limited by the nature of the participants' pervasive mental health illness and medication that firstly impacted on their potential inclusion in Phase 1 and 2, and then eventual identification as a information-rich participant for this Phase. In addition, mental health status and medication often impacted the participants' communication ability and skills. Furthermore, most of the participants had limited education. The team was interested in capturing the participant's initial responses to the survey questions, and to prevent the risk of second interview process and recall bias, the participants did not have the opportunity to review or edit their responses after the interview was completed and electronically uploaded. The non-validation of responses may have limited the findings, however, the interviews were conducted in a circuitous manner that enabled continuous confirmation of responses, and all participants made sufficient statements for this study.

## Conclusion

Intimate relationships have a protective effect on the quality of life and recovery (64), yet sexual health and sexuality in forensic patients have received scant attention, and there have been no large-scale studies focused on forensic community mental health patients concerning their sexual wellbeing. Previous studies on this topic focused on inpatient settings (36, 84, 85). Therefore, the current study aimed to qualitatively examine participants' sexuality and sexual experiences with major mental illnesses treated under the legal framework of a forensic order in a community setting. The knowledge gained from this study can be used to develop and implement evidence-based interventions to address the sexual health needs of community forensic mental health patients.

“*The greatest and most healing service that can be offered to people with psychiatric disabilities is to treat them with respect and honor them as human beings. This means honoring us in our full humanity, including our sexuality and our desire to love and be loved.”* (1)

This poignant quote should serve as a reminder for those of us in the service of treating patients with enduring mental illnesses. Although there are major barriers for forensic mental health patients to achieve their ideal sexuality and romantic lives, these barriers can be addressed to reduce their impact and improve their quality of life. Our findings suggested multiple ways mental health teams can improve their patients' sexuality and sexual experiences and demonstrated that many participants would be receptive to this support.

Mental health teams need to create a safe space for their patients to come forward with sexual health problems without feelings of judgment or shame. Review of sexual health should be a standardized part of a mental health review, and management of sexual health issues should form part of clinical care and it is an essential component in overall wellbeing. Therefore, it is essential to have a clear understanding of sexuality and sexual activity in people with major mental illness to identify areas where this vulnerable population can be supported in achieving optimal sexual health.

## Data Availability Statement

The datasets presented in this article are not readily available due to patient privacy restrictions. Requests to access the datasets should be directed to s4454530@student.uq.edu.au.

## Ethics Statement

The studies involving human participants were reviewed and approved by Royal Brisbane and Women's Human Ethics Committee, Queensland Health. The patients/participants provided their written informed consent to participate in this study.

## Author Contributions

EB conceived and developed the method and undertook the data collection for this research. DN was involved in the interpretation of the qualitative analysis. EB and DN wrote the initial manuscript. AR and EH edited the manuscript. All authors have approved the final manuscript.

## Conflict of Interest

The authors declare that the research was conducted in the absence of any commercial or financial relationships that could be construed as a potential conflict of interest.

## Publisher's Note

All claims expressed in this article are solely those of the authors and do not necessarily represent those of their affiliated organizations, or those of the publisher, the editors and the reviewers. Any product that may be evaluated in this article, or claim that may be made by its manufacturer, is not guaranteed or endorsed by the publisher.
