# Supplementary material for: A Qualitative Study on Sexuality and Sexual Experiences in Community Forensic Mental Health Patients in Queensland, Australia
Source: Front Psychiatry. 2022 Mar 31;13:832139. doi: 10.3389/fpsyt.2022.832139 (PMC9008211; doi:10.3389/fpsyt.2022.832139)
Supplement: Supplementary file 1 [file Data_Sheet_1.pdf]

Phase 3: Let's talk about sex

These questions have been developed in collaboration with a senior forensic psychologist and consumer advisor.

Introduction
I am continuing the research into the experiences of people engaged with mental health services and their sexual experiences. You might remember that you have answered similar questions during previous phases of the study.

We will cover some topics that could be perceived as difficult or sensitive. If you do not wish to answer the question, just say that you want to move to the next question.
I will be using words that clearly describe acts around sex and I encourage you to use whatever words you feel comfortable with.

Questionnaire

Can you please tell me about your current sexual life?

- **Is your current sexual life and experiences what you want it to be?**
- **Are you happy and satisfied with your sex life?**

Prompts – in terms of gender identity, sexual identity, male and female partners, difficulties, emotional experience, masturbation, orgasm achieved, pornography, non-coital experiences, sex work, sexual coercion, contraception use, sexually transmitted diseases, sex and the internet (use of porn hub, red tube, peer to peer network sharing, paid cam shows, erotic literature), frequency.

Can you please describe your ideal sexual life?
Prompt – in terms of gender identity, sexual identity, male and female partners, difficulties, emotional experience, masturbation, pornography, non- coital experiences, sex work, sexual coercion, contraception use, sexually transmitted diseases, sex and the internet.

Have there been any barriers preventing you from reaching your ideal sex life? What are they?
Prompt - in terms of physical, emotional, social, practical, medical

How do you think your mental health team can support you in achieving your desired sex life?
Prompt - in terms of biological, psychological, social, cultural, religious.

How would you feel if questions around sexual health are asked in history taking or as part of reviews?

Do you think Mental Health Services have a role to play in supporting your sexual health and wellbeing?

Closing statement:
Thank you for your participation today. You can talk to your Mental Health Services team, and Forensic Liaison Officer should you need further support or clarification around these topics.
